# The challenges of change processes for nurse leaders—a qualitative study of long‐term leaders' experiences over 25 years

**DOI:** 10.1002/nop2.1781

**Published:** 2023-04-30

**Authors:** Marianne Frilund, Lisbeth Fagerstrøm, Frøydis Vasset

**Affiliations:** ^1^ Department of Health Sciences in Ålesund, Faculty of Medicine and Health Sciences Norwegian University of Science and Technology Ålesund Norway; ^2^ Rector of Åbo Academi University in Vasa, Professor in Caring Science Faculty of Education and Welfare Studies Åbo Akademi University Vasa Finland; ^3^ Department for Health University College in Molde Molde Norway

**Keywords:** change processes, leadership, nursing

## Abstract

**Aim:**

This study aimed to map what experiences nurse leaders have encountered concerning the change work that political decisions and reforms have created within the healthcare sector in the last 25 years.

**Design:**

A qualitative design with a narrative approach was used.

**Methods:**

A qualitative study involved individual interviews of eight nurse managers from Norway and Finland with more than 25 years of experience working in specialist and primary healthcare fields.

**Results:**

Two main categories were observed: experiences of organizational challenges and experiences of personnel‐administrative challenges. The first main category included two subcategories: A: historical experience with culture and challenges in health services and B: historical experience with mergers and using welfare technology in health services. The second category included the following subcategories: A: historical experience of job satisfaction for leaders and employees and B experiences with interprofessional collaboration in health services.

## 1 INTRODUCTION

Healthcare services in Nordic countries have undergone considerable organizational changes, which have correspondingly challenged the role and status of nurse leaders at all levels of health services (Hafsteinsdóttir, 2019; Storbjörk et al., 2021). By improving relationships between different professions and organizations and through motivating and responsible change processes, positive patient and professional outcomes are activated.

The Ministry of Social Affairs and Health is responsible for the development of health services in Finland, while the Norwegian Directorate of Health is responsible in Norway. The need for changes is often a result of political decisions that are published in legislation, recommendations and different health reforms (White Paper, 2008‐2009). These political decisions in both Finland and Norway have had a major impact on operational and professional leadership and management with major change processes as a result.

The ideology of New Public Management (NPM), digitization and the priorities of open healthcare versus hospital care is noteworthy because of the requirements for organizational and personnel changes their implementation demanded (Nylenna, 2020; Orvik, 2015). The ideology of NPM became common in healthcare organizations in Nordic countries in the 1990s. The nurse leader's role in the organizations was changed from the role of a clinical expert to a more non‐clinical one. Leadership became largely based on strategic management, administration, development of content and work around training of the staff. Furthermore, non‐clinical leaders were no longer engaged in direct contact with patients or played any active role in patient care, as clinical leaders did (Feitosa et al., 2021).

Other changes over the 25 years include the digitization and the priorities of open health care versus hospital care. The implementation of new health technology has also initiated a change process and has the potential to alter methods of working, organizing work and power relations in health organizations (Vaagan et al., 2021). Digitization has been (and still is) a major challenge for health organizations (Vaagan et al., 2021). To create an alliance to ensure proper health care, leaders are required to develop leadership competencies that differ from the typical criteria associated with clinical and scientific excellence (Vasset et al., 2022; Ylitörmänen et al., 2019).

Leaders at all levels in health services experience major change, with challenges for both leaders and employees. Organizations merge into large units, with wide geographical spread and different cultures. According to Salmela et al. (2012) and Vasset et al. (2023), nurse leaders promote change by leading relationships, processes and culture and by using supportive, reflective and culture‐bearing leadership that permits the realization of genuine and sustainable changes (Salmela et al., 2013, 2017; Vasset et al., 2022, 2023).

Leadership, which emphasizes innovation, creativity, competencies and the participation of employees in strategic planning, has resulted in the knowledge of rules or bureaucratic procedures becoming less relevant. Healthcare teams often work in uncoordinated environments, thus resulting in the duplication of processes, and increased costs and workload. Fast‐paced, demanding working environments have led to increased medical errors and threatened patient safety. For decades, hierarchies between nurses and medical and financial leaders have existed. The role of nurse leaders has often been unclear (Yukl, 2019). In contrast to nurses, physicians are formal leaders who engage in direct contact with patients, and the role of nurses has been to indirectly create the conditions required for reasonable care (Huikko‐Tarvainen, 2022; Vasset et al., 2021, 2022). In order to lead change processes, an effective leader has to use different relationship‐oriented behaviours to build commitment, mutual trust, collaboration, and identification with the team. Moreover, the leader builds and maintains a network of cooperative relationships with outsiders who are a valuable source of information, assistance and political support, and who also forge a coalition of internal and external supporters (Booher et al., 2021; Hemberg & Salmela, 2021; Salmela et al., 2017).

The purpose of this study was to map what experiences nurse leaders have encountered in connection with the change work that political decisions and reforms have created within the healthcare sector in the last 25 years.

This case study is a qualitative study based on individual interviews among eight nurse leaders from two countries, Finland, and Norway.

## 2 BACKGROUND

Earlier research was conducted with the help of a systematic database search by using the following keywords: change processes, nurse leader and challenges in health services. Several concepts were found to be common to interprofessional and inter‐organizational collaboration, such as communication, trust, respect, mutual acquaintanceship, power, patient‐centredness, task characteristics and environment (Almås et al., 2018). Other concepts are of particular importance either to inter‐organizational collaboration such as the need for formalization and the need for professional role clarification or to interprofessional collaboration such as identifying the role of individuals and teams. The promotion of inter‐organizational collaboration was observed to face greater challenges, regarding matters such as the achievement of a sense of belonging among professionals when differences exist between corporate cultures, geographical distance, the multitude of processes and formal paths of communication span style (Karam et al., 2018).

Employees' reactions to changing processes can vary depending on the desire for change. Previous research has described employees' reactions to change in terms of threats, job satisfaction, problems and solutions (Holmström et al., 2021; Nilsen et al., 2016). Approximately 40%–80% of traditional change efforts result in failure, as reported by Nilsen et al. (2016). This failure may be due to the existence of multiple frameworks for understanding the types and the nature of change and a lack of conceptual clarity regarding the tasks of leading, managing and directing change. However, many change initiatives fail because of unfocused and insecure management and due to a lack of systematic project management (Holmström et al., 2021).

The effective nurse leader leads interpersonal relationships through an ethical approach of respect, trust and concern to preserve a trustworthy relationship with co‐workers. Moreover, the nurse's leader is an interactive team player (Gjellebæk et al., 2020).

Changing processes benefit from effective nurse leaders who lead through an ethical leadership style characterized by respect, trust and consideration (Hemberg & Salmela, 2021; Holmström et al., 2021).

Leading change processes are not a new concept for nurses; however, the character of such changes is complex, and the processes are faster‐paced than they were 25 years ago.

Collaborative practice has been highlighted as a necessary aspect of implementing change processes and addressing health problems in a complex municipality (Almås et al., 2018; Guraya & Barr, 2018; Vasset & Almås, 2017). Employees and leaders in health organizations must collaborate in an individual organization and among different organizations, countries and professions. These principles are similar across health professions and are often enumerated in terms of interprofessional collaboration, sharing, partnership, interdependency and power (Green & Johnson, 2015; Hove & Vasset, 2020; Karam et al., 2018; Lindquist, 2018).

A study by Vasset et al. (2022) demonstrates that nurse leaders have shifted from serving as clinical experts to performing purely administrative work. One of the most important tasks for nurse leaders is to develop their skills to lead and manage all the challenges that the change process requires (Hemberg & Salmela, 2021; Salmela et al., 2017).

The research question is as follows:

*What challenges have nurse leaders had in terms of organizational change processes in their work over 25 years?*

## 3 METHODS

### 3.1 Design

In this study, we employed a qualitative narrative design (Kvale & Brinkman, 2015; Polit & Beck, 2017), which has been identified as being the most suitable method for exploring experiences and opinions and for gaining a comprehensive understanding of a leader's work history. The narrative design describes life in its entirety by recapitulating past experiences and matching a verbal sequence of causes to a series of events (Polit & Beck, 2017). The term ‘history’ is equivalent to ‘narrative’, thus indicating that the narrative is the primary form in which humans live.

### 3.2 Sample

The study included participants from primary and specialist health services in Norway and Finland. The leadership role of the participants was largely based on strategic management, administration, development of content and work training. All participants had formal leadership and management position. The leaders' titles differ between the countries. In this study, we chose to use the term ‘nurse leaders’ synonymously to ‘head nurses’ or ‘chief nurses which comprises that they were responsible for resource allocation, administration, staff and service quality at an intermediate level. Prior to the data collection, specialists and primary health service leaders were contacted and informed of the study via e‐mail. The intention was to recruit participants with long‐term and profound experiences as nurse leaders in the context of healthcare services or specialist care. They should also have experience in leading change processes. The first eight nurse leaders who accepted the invitation to participate in the study were included, that is four nurse leaders from primary health services and four nurse leaders from specialist health services, for a total of four nurse leaders from each country. Table 1 shows a description of the informants’ formal education, higher education, organizational affiliation, title and nationality.

**TABLE 1:** Description of the sample in this study.

Code for informants^a^	Formal education	Higher education	Organization		Current title	Nationality
1 N	Nurse	Leadership—master's degree	Specialist service	Health	Head nurse	Norwegian
	Face to face					
2 N	Nurse	Leadership, economy	Primary health service		Head Nurse	Norwegian
	Face to face					
3 N	Nurse	Leadership Guidance pedagogy	Primary health service		Head Nurse	Norwegian
	Face to face					
4 N	Nurse	Leadership—master's degree	Specialist service	health	Head Nurse	Norwegian
	Online					
5F	Biomedical laboratory scientist	Performance management. Master's degree and PhD in health sciences	Specialist service	health	Head nurse	Finnish
	online					
6F	Nurse	Master's degree in management; Lean education, expert nurse	Specialist service	health	Head nurse	Finnish
	online					
7F	Nurse	Master's degree in management. Expert nurse	Primary health service		Head nurse	Finnish
	Online					
8F	Nurse	Expert nurse; Master's degree and PhD in nursing sciences	Primary health service		Chief nurse	Finnish
	Online					

*Note*: 1 F = the first informant from Finland; 2F = the second informant, etc.

^a^ 1 N = the first informant from Norway; 2 N = the second informant, etc.

Prior to the data collection, specialists and primary health service leaders were contacted and informed of the study via e‐mail. The intention was to recruit participants with long‐term and profound experiences as a nurse in the context of. In other words, the informants were located through recommendations from well‐known healthcare professionals. The participants were required to (1) have full‐time jobs, (2) have been nurse leaders for approximately 25 years and (3) speak Norwegian or Swedish.

### 3.3 Data collection

Data were collected through individual interviews with an open interview guide. The same number of interviews was carried out in both Norway and Finland. At the beginning of the data collection process, three interviews were carried out face to face; but due to the COVID‐19 pandemic, some of the data was collected virtually. All the interviews were audiotaped, with the written permission of the informants. Additionally, the interviews lasted approximately 1½ h each and were completed in the spring–summer of 2020. The focus of the narrative interviews was on the depth of the conversations.

Background data are presented in Table 1. Table 1 highlights the level of education of the nurse leaders and demonstrates variation between the Nordic countries, which can possibly be explained by how nursing science as an academic discipline has developed in different countries (Hafsteinsdóttir, 2019; Vasset et al., 2021, 2022). The main question the participants were asked was, which challenges they had experienced during the changing processes.

The analysis process followed the four steps of text condensation suggested by Malterud (2017).

(1) Firstly, the transcripts were read by the first author and third authors to obtain an overall impression of the respondents' experiences. (2) Secondly, a detailed reading of the transcripts was performed to identify relevant descriptions of experienced challenges during the change processes. (3) Thirdly, the content of meaningful units was abstracted into concepts and coded into categories and subcategories. (4) Finally, the essence of each category and subcategory was summarized and used as a foundation for the result section.

### 3.4 Ethical considerations

The Research Ethics Committee's approval for this study was obtained from the Norwegian Social Science Data Services. Informed consent was obtained from all the respondents in accordance with the rules stipulated by the Helsinki Declaration. We conducted eight narrative interviews with nurse leaders in healthcare institutions. Moreover, the same number of interviews was conducted in both Norway and Finland. The interviews were open ended to provide the informants with the opportunity to convey a complete picture of their careers. Dictaphones and virtual platforms were used during the interviews. Furthermore, the focus of the narrative interviews was on the depth of the conversations. After eight interviews had been conducted, we could observe similarities and differences in the informants' experiences. However, we could also observe clear themes emerging based on the interpretation and analysis processes.

The participants were informed that they could withdraw from the interview in 4 months but before the writing of the articles began. The participants had been numbered and could be identified by the authors. The first author has an overview of the informants from Finland, and the third author has an overview of those from Norway. The participants were provided with both written and verbal information regarding the purpose of the project. However, it was in the researchers' interest to protect the identity of the informants as far as possible. The audio tapes and data about the informants were kept apart. The informants were informed of this and asked to give informed consent to the accepted conditions before the survey.

## 4 RESULTS

The analysis process generated two main categories: (a) organizational challenges and (b) personnel‐related and political challenges. Table 2 shows the main categories and their subcategories and example quotations to explain the interpretations of the transcriptions. Each category with subcategories is presented as a synthesis formulated across the interviews and illustrated with examples of quotations.

**TABLE 2:** Change process in health services.

Categories	Subcategories	Direct quote from informants
Experiences of organizational challenges	A: *Historical experience with culture, challenges in health services* B: *Historical experience with mergers and the use of welfare technology in health services*	The patients were in hospital when they were ill, and we wrote in patient‐journals. Do we think we had more time for patients? It was mostly older people who received help this first years. It was the doctor who was the most important in the hierarchy, at that time. Knowledge began to increase; other digital opportunities came already in the 80s and 90s. Lead yourself; you must seek evidence yourself: you must be up to date on what is happening. The merger came, the Cooperation Reform came, the Hospital Reform came, it became a health trust. New reforms–We spent many times creating a common culture, then we were merged with AAA hospital. We had cooperation problems from the first moment. Distance management is a challenge. Management meetings in NNN city, it was a challenge. Can feel a little forgotten. Various welfare technologies are used in homes, less so in nursing homes. A lot about security alarms and GPS in shoes, e‐locks like to see a change for the better. I'm a problem solver. Then, we started with RAFAELA when I got some results, I had it as a matter, and I showed and told.
Experiences of personnel – administrative challenges	A: *Historical experience of job satisfaction for leaders and employees in health services* *B Experiences with interprofessional collaboration in health services*	Being motivated to work for change depends on one's own goals and expectations that the change must bring with it We worked interprofessional with physiotherapist, occupational therapist, speech therapist and doctor. That was what appealed me to be leader of many strong professions. I believe in teamwork. I have always been the one who fights. The health platform will enter into force in 2021. What gives strength as a leader, you have your own personal weaknesses and strengths. If you think that you belong to a management group that prepares matters for a committee or board, you must learn to recognize what is politically possible. I think I am become quite good at reading what's going on. That you see that on the “nose position.” You must listen carefully and read what the politicians talk about that you understand what they mean and see what is possible. Then, you must learn to take advantage of the opportunities when they come. That you take up proposals at the right time. Sometimes you must bargain for your ideals. One must realize the economic and political realities and try what is possible.

### 4.1 Experiences of organizational challenges

The category related to organizational challenges was divided into two subcategories: (a) culture, quality and economic challenges and (b) geographical distance and technology challenges.

#### 4.1.1 Historical experience with culture, challenges in health services

The informants highlighted the concept of culture and its significance to change processes. The informants talked about organizational cultures that vary across occupational groups, departments and care units, such as primary healthcare units versus specialist healthcare units. The informants stated that the concept of culture is not clear to everyone. Organizational culture can be a foreign concept to some individuals and must be repeatedly explained and discussed at various levels and with respect to change processes. The informants emphasized the importance of a shared understanding of the concept of culture and what it means for a potential new organization. The common understanding of the concept of culture can be a success factor for the entire change process, as highlighted by the informants.

I concluded that you lead changes through the employees, the processes, and the culture. Later, it has emerged in our organization that you change the culture, which is especially important. If you are going to make a change, you must be aware of the culture and the importance of what culture means (F8).

The establishment of a standard set of values may take up to 4–5 years, some of the informants stated. They continued the discussion by stating that if the change process involves a statistically significant change or a central organizational change process, the leaders must spend considerable time to create common ethical standpoints and values.

The participants shared input about the fact that employees compared their current situation with the situations they experienced prior to the beginning of the change process. According to the participants, the focus on arrangements and the person's responsibility for the change process can be complicated. New goals can be created, by the employees and their leaders, but despite that the staff needs to continuously be reminded about the deals that have been made earlier. Moreover, employees' needs for these alterations affect their daily work, goals and strategic planning. The following quotations highlight the complexity of change work and the challenges faced by the leader.

You go back all the time to the fact that we agreed on this last year and two years ago, and now we have come this far. Now there have been delays. You inform and inform, and when it comes thus far, it is said that we have not received any information (F5).

In other words, these activities are time‐consuming processes for repeated information and support. Culture also pertains to diverse ways of collaborating with professional groups and organizations. The participants referred to previous leadership models that were strongly characterized by hierarchical thinking. Doctors traditionally made decisions, and doctors had traditionally been regarded as leaders. In this scenario, the participants had experienced the implementation of NPM. Moreover, the organizations were divided into result units, and nurse leaders had to abandon responsibility for decisions. The informants from Finland identified two equivalent “lines” (one line for medicine and one line for nursing). For other participants, nursing was included in the medical leadership (Norway). By using two equivalent lines, the participants noted that they had strengthened the position of nursing in the organization. Moreover, other nurses continued to express the opinion that doctors shape the vision and goals of healthcare organizations, whereas nurse leaders played subordinate role.

I thought I would have the opportunity to develop this, it gets better and better all the time. It is the hierarchy that comes in. A few doctors want to be involved in the development, but far from all. If we had the old leadership model, we (nurse leaders) would probably have even more difficulties (F6).

The intention is that nursing and medicine should be equivalent, but it depends on the person, how you want to lead (N4).

In the 1980s, new models for treatment and caring were developed, which led to a need for new organizational and care models (i.e. a new nursing ideology or culture). The processes of nursing and caring would be developed in accordance with the patients' needs and adapted to contemporary norms and value systems, one informant stated.

The mindset that one must look after the client's needs and opportunities adapts the business to support the client. It taught me an incredible amount to be able to follow the rehabilitation process of a client (N4).

In addition to cultural changes, the informants also emphasized the need for quality insights into financial awareness. Patient or user perspectives were strengthened, which was an experience shared by all participants. Additionally, the quality of nursing was highlighted by the informants. Changes occurred that featured an emphasis on outpatient care versus institutional care. Moreover, all the participants highlighted the importance of high‐quality nursing and care. Research, education and evidence‐based praxis were some of the areas in nursing to which nursing leaders were required to pay attention throughout their careers.

Then, it is all nurses who perform clinical work with this evidence. To seek evidence, it must be evidence‐based. I no longer approve of something like this that we have always done. It is this more innovative thinking that is more visible in care work today. We are knowledgeable that we have research results. It is a strength of my department leaders today; they are probably active (F5).

Based on the data, it appeared that political decision‐making and economic realities often constitute motivations for change rather than aspects related to culture or quality, which professional managers cannot ignore. However, simultaneously, the economic reality does not exclude opportunities to work to provide high‐quality services.

Then, it is economical. You have the money you must use that the municipalities give, and we always exceed it, but it is probably a constant struggle with finances (N2).

#### 4.1.2 Historical experience with mergers and the use of welfare technology in health services

Changes of various kinds have occurred throughout the participants' careers, but these changes are expected to occur more quickly and become more significant than the participants' discussions indicate. At the beginning of their careers, nursing leaders were leaders of small organizations, in which context they leader could oversee the work activity in detail. Today, organizations have expanded and have become too large and too complex to ‘own’ all the ongoing projects.

The merging of different hospitals is a substantial change that results in extraordinary challenges for leaders and employees. Legislation and national recommendations in Finland and Norway have highlighted the need for larger units, which would be more cost‐effective and increase the quality of services.

The merger came, the Cooperation Reform came, the Hospital Reform came, and it became a health trust (N1).

An obvious problem that emerged in the data material was the sizeable geographical spread of the organizations, which complicates the developmental processes of the new organization. Contact in the organization often takes place via digital platforms, which is an approach that the informants experienced as being challenging with respect to contact creation and interaction.

New reforms–We spent a great deal of time creating a common culture and merged with AAA Hospital. This merger was not life changing. The problem was that we had collaboration problems from the first moment (N4).

One participant described her experience as follows: Sometimes, you feel like you are just a picture on the wall during Skype meetings (N4).

Another informant continued as follows:

Management meetings in NNN hospital were a challenge. I felt that I was a little forgotten and that distance management was a challenge. They became a battle about resources, and that was unsatisfactory. When you get a wider area, you come to the realities of the budget (N4).

I have also had contact with politicians in the leadership role, which was not the case at the departmental level (N4).

Welfare technology was incorporated into the organizations quite early in the participants' careers. The participants noted that knowledge began to increase, and other digital opportunities were already available, in the 1980s and 1990s. Some participants perceived introduction and training in the use of various devices (such as computers) as deficient. Moreover, the respondents described everyday life as being overtaken by problems created by computers. Other nurses viewed IT technology as being an aid to the acquisition of new knowledge, not merely for nursing documentation.

Nevertheless, the introduction of welfare technology created a need for new models and routines in nursing. Several informants noted that in terms of finances, welfare technology was not particularly profitable. They discussed licences, training, year‐round service, repairs and new parts. Additionally, there was always some factor that did not work as it should have.

Participants noted that it was mainly municipalities that knew which welfare technology could be used at the patients' homes.

Various welfare technologies are used in homes, but less so in nursing homes. A lot about security alarms and GPS in shoes, e‐locks. I like to see a change for the better (N2).

To ensure more efficient health care, get the devices and new computer systems without much training. I have reported this back to the director (N4).

### 4.2 Experience of personnel administrative challenges

The second main category of challenges was related to personnel administrative challenges. The main category was related to two subcategories: (a) job satisfaction and motivation and (b) political and interprofessional collaboration.

#### 4.2.1 Historical experience of job satisfaction for leaders and employees in health services

The participants experienced reluctance to change. This reluctance is often rooted in various types of fears. Staff members are anxious about losing their jobs, the inability to cope with new tasks and having to move to another workplace that features new, unknown routines and people. Moreover, the participants noted that employees perceived mergers as threats. One frequently raised question was ‘Whether I will be allowed to keep my job. Will I get new tasks that I do not master?’ A challenge for nursing leaders pertained to the creation of security and trust and the task of listening to and informing employees. Furthermore, participants noted that displaying a cheerful outlook towards change work was a challenge.

Being motivated to work for change depends on one's own goals and expectations that the change must bring with it (F6).

As brought out by the informants, mergers among organizations resulted in changes in terms of leadership responsibilities. The leader was given greater responsibilities and additional employees to manage. Additionally, the personal relationships between leaders and employees were weakened. The leader did not ‘know’ their staff in the same manner as before. Moreover, the informants felt they became more distant from clinical work and began to serve as a full‐time administrator.

It was a merger of units, which met with resistance. I must listen to them, and there was a concern, will I be without work? I must move there (F5).

Several informants highlighted the fact that you must listen to employees, engage in discussion with them and motivate them to perceive new opportunities. Some employees shared that they did not always know if they could trust that they would be well cared for during the change process.

Some have difficulty making the change, depending on how you are as a person. However, you must understand them too. If they have been at a small place, they will be afraid to come to a larger place, afraid of not being able to do it, if it is too demanding, do I have the required knowledge, or will I be forgotten (F5).

A leader's task is to address fears and anxiety and to motivate change work. At present, leaders have fewer opportunities to inform employees of changes and to discuss that topic with the employees. The larger and more complex an organization grows, the more insecurity emerges among employees. The participants noted that they often did not know each other, which could be a challenge. Moreover, the workplace was viewed as pleasing when employees exhibited certain traits. As one informant noted, ‘they must like challenges and those who are going to start here must be independent and not focus on details all the time’ (N1).

A prerequisite for successful change is that employees must feel as if they are involved in the change process and must understand why a change is necessary. Furthermore, attitude change requires time and many extensive discussions.

#### 4.2.2 Experiences with interprofessional collaboration in health services

Some participants expressed the notion that being the leader of an interprofessional team was appealing to them. ‘We worked interprofessional with physiotherapists, occupational therapists, speech therapists and doctors in this department. It is a challenge to be an interprofessional leader and a leader of many vital professions’ (N4). The change affects people in both positive and negative ways. A leader should first ensure that employees are aware of the reason for such a change. That most of the changes were initiated by politicians without sufficient knowledge of the consequences that a decision had for the organization and the employees, was a common experience among the informants.

If you believe that you belong to a management group that prepares matters for a committee or board, you must learn to recognize what is politically possible one informant stated. I think I have become quite good at reading what is going on (F8).

The informants stated that the leader must listen carefully and read what the politicians talk about to understand what the politicians are discussing and to determine what is possible. Afterwards, the leader must learn to capitalize on the opportunities when they arise and accept proposals at the right time.

One must realize the economic and political realities and try what is possible (N5).

Why is it important that units are merged, the organizational models are developed; what is the utility of the change? (F6).

These quotes emphasize the idea that important questions need answers, and the questions may not be easy to answer.

## 5 DISCUSSION

Based on the two main categories, experiences of organizational challenges and experiences of personnel administrative challenges with subcategories, analysis and interpretation of the findings was carried out. The informants highlighted culture, quality of services and economics as essential issues that are relevant to change processes. In addition to cultural changes, the informants also emphasized the need for *quality insights* and *financial awareness*. The second category is related to job satisfaction and motivation and to political and interprofessional collaboration. Job satisfaction, motivation and political and interprofessional collaboration have been highlighted in earlier research (Salmela et al., 2017; Storbjörk et al., 2021).

### 5.1 Organizational challenges from a historical perspective

The results confirmed that organizational challenges have been a reality throughout the careers of nurse leaders. New reforms have occurred, new welfare technology has been developed and mergers of health institutions have occurred because of political intentions and goals. Culture, quality and economy have been relevant themes in connection with the changes that have been relevant in recent decades (Huikko‐Tarvainen, 2022; Nylenna, 2020).

In the early 1990s, these changes frequently pertained to new care models, service offerings and leadership issues. Political leaders have given considerable attention to quality assurance models, efficiency and productivity (Gjellebæk et al., 2020; Karam et al., 2018). However, there is an obvious risk that such a focus on efficiency and productivity may become guidelines for health care, potentially at the expense of the well‐being of employees and users. Changes occurred that emphasized outpatient care rather than institutional care. (Hemberg & Salmela, 2021). Additionally, all the participants highlighted the importance of high‐quality nursing and caring. Research, education, collaborative praxis and evidence‐based praxis were some of the areas within nursing to which nurse leaders were required to pay attention throughout their careers (Vasset et al., 2022, 2023).

The informants noted that the concept of a culture is not self‐evident, and they spent a great deal of time developing a common understanding of the concept. A shared understanding of the concept of culture, economy and quality of service, and what this understanding means for the organization, can be a success factor for a new organization (Byrkjeflot & Jespersen, 2014; Salmela et al., 2013; Vasset et al., 2022). According to Salmela et al. (2012), nurse leaders inaugurate change by leading relationships, processes and culture and via supportive, reflective and culture‐bearing leadership aimed at the realization of genuine and sustainable changes. These claims were supported by this study.

Change processes have created complex organizations due to cultural differences across organizations in terms of quality and economic realities (Gjellebæk et al., 2020; Guibert‐Lacasa & Vázquez‐Calatayud, 2022). Participants from Finland and Norway highlighted the shared culture of the organization, the optimal quality of care, treatment and a responsible economic policy focused on the patients' needs. The change processes created complex organizations, difficulties for personnel and political challenges. The ultimate purpose of public services is to produce what is beneficial to citizens (Nylenna (2020); Virtanen & Stenvall, 2010). One challenge to the provision of excellent service may be the geographical realities resulting from mergers between organizations. It is difficult to lead changes without conducting a meeting, as the informants stated. The informants felt as if they were ‘pictures on the wall’ in the absence of face‐to‐face contact. Additionally, the constant need for change is a reality, which is partially due to changes both within and outside of organizations, not merely due to political recommendations and reforms, which have become accelerated in recent decades. Feitosa et al. (2021) highlighted the concept of ‘political skills’. Specifically, a leader in health care must collaborate with different people from different professions with respect to decision‐making and strategic planning. Political skills can be defined as the ability to adapt to changing situations and the behaviour of others to align with personal or organizational goals (Bernstrøm, 2014;Feitosa et al., 2021; Nordquist & Grigsby, 2011). Traditionally, leadership tasks in the public sector have been rewarded when leaders display a strong work ethic and clinical competence, and less attention has been given to leadership competencies (Huikko‐Tarvainen, 2022; Nylenna, 2020).

Effective leadership is critical for optimizing the cost, accessibility and quality of health care according to Hafsteinsdóttir (2019). Interprofessional collaboration, political skills and staff motivation are essential to accomplishing healthcare goals (Almås et al., 2018; Feitosa et al., 2021; Hafsteinsdóttir, 2019; Hove & Vasset, 2020; Nylenna, 2020; Virtanen & Stenvall, 2010).

### 5.2 Historical experience of personnel administrative challenges

In the modern world, health organizations have expanded, and organizations have become excessively large and complex.

When change works in health organizations, motivation, goal formulation, information, communication, fear, insecurity, participation and cultural interactions are vital elements according to the literature and administrative research (Byrkjeflot & Jespersen, 2014). Economic realities, efficiency and quality often describe the need for change. Research has indicated that 40%–80% of traditional change efforts result in failure (Nelson‐Brantley & Ford, 2017). The leading of change processes (which occurs more frequently in health organizations) is experienced as an unfortunate and almost overarching responsibility by leaders and employees (Huikko‐Tarvainen, 2022). The participants referred to previous leadership models that had strong roots in hierarchical thinking. From this perspective, doctors have a long historical tradition as leaders (Huikko‐Tarvainen, 2022), as noted in the studies by Vasset et al. (2021, 2022, 2023).

The nurse leaders who participated in this study were satisfied with their work. but the employees perceived all mergers as threats. This situation is difficult because employees do not always know whether they will keep their jobs. Health care has been transformed from a locally controlled effort to a highly fragmented national system facing unpredictable changes, which is a point that the informants discussed in the study (Feitosa et al., 2021; Nordquist & Grigsby, 2011).

Some delays occur during the change process; specifically, leaders inform employees, and the employees feel as if they have not received any information. Salmela et al. (2013) emphasized the importance of repeating information several times. Research has also highlighted reactions to change on the part of management as a process that progresses through several phases (Guibert‐Lacasa & Vázquez‐Calatayud, 2022; Mignonac, 2008).

Threats, job satisfaction, problems and solutions were mentioned by the participants. There is reason to believe that job satisfaction varied slightly. Too many mergers with diverse cultures are difficult for employees. Additionally, a leader should first ensure that employees are aware of the reason for a change. Political knowledge is a requirement for a leader in a healthcare organization in order to foster different entities.

People from different backgrounds plan the work process in the context of everyday work to allow them to meet each other. Researchers have highlighted this fact (Bernstrøm, 2014; Feitosa et al., 2021; Nordquist & Grigsby, 2011). According to Nylenna (2020), NPM is intended to distinguish between political and professional leadership, highlight results and foster active communication with political and professional leaders. Several informants noted that they had become quite good at researching what was occurring regarding employees' feelings and new political guidelines. The changes led to nurse leaders being given leadership responsibilities for other areas associated with the profession (such as responsibility for employees in other locations or other hospitals).

## 6 CONCLUSIONS

The challenges highlighted in this study were organizational and personnel changes occurring over several decades. However, new reforms have been implemented, new welfare technology has been developed, and mergers of healthcare institutions have occurred, which were often due to political intentions and goals. The implementation of change processes to ensure effective health care with high quality takes time.

Following a merger, it is difficult to create new organizations with interprofessional teams that are distant from one another over a short period of time. Mergers that blend different departmental and institutional cultures are tiring for employees. Moreover, interprofessional collaboration, political skills and employee motivation are essential for achieving the goals of the healthcare system. Political knowledge is essential for leaders in the healthcare system because they must collaborate with people from different backgrounds and plan the work process. Furthermore, the NPM intended to distinguish between political knowledge and professional leadership.

## 7 LIMITATIONS

The data referenced in this study were collected during the pandemic, thus making face‐to‐face interviews unfeasible. Additionally, only eight participants were included in the study, but our opinion is that a certain degree of saturation is apparent in our results. Another weakness of the study is the fact that the survey group from Finland consisted entirely of Swedish‐speaking leaders. We may have been able to account for more nuance in our results if both language groups had been represented.

## AUTHOR CONTRIBUTIONS

Frilund has the main responsibility for the article. Vasset (lives in Norway) is responsible for the Norwegian data material. Frilund and Fagerstøm live in Finland and are therefore responsible for the Finnish data material. The introduction, background and analysis were carried out by Vasset and Frilund. All the authors read and approved the submitted article draft.

## FUNDING INFORMATION

There has been no funding.

## CONFLICT OF INTEREST STATEMENT

There are none to declare.

## ETHICS STATEMENT

The Research Ethics Committee approval number and the name of the review board that approved the study of the Norwegian Centre for Research Data (NSD) (no. 750316).

## Data Availability

Not applicable.
